# Comparison of Ranson, Glasgow, MOSS, SIRS, BISAP, APACHE-II, CTSI Scores, IL-6, CRP, and Procalcitonin in Predicting Severity, Organ Failure, Pancreatic Necrosis, and Mortality in Acute Pancreatitis

**DOI:** 10.1155/2013/367581

**Published:** 2013-09-24

**Authors:** Ajay K. Khanna, Susanta Meher, Shashi Prakash, Satyendra Kumar Tiwary, Usha Singh, Arvind Srivastava, V. K. Dixit

**Affiliations:** ^1^Department of General Surgery, Institute of Medical Sciences, Banaras Hindu University, Varanasi, Ultra Pradesh 221005, India; ^2^Department of Pathology, Institute of Medical Sciences, Banaras Hindu University, Varanasi, Ultra Pradesh 221005, India; ^3^Department of Radiodiagnosis, Institute of Medical Sciences, Banaras Hindu University, Varanasi, Ultra Pradesh 221005, India; ^4^Department of Gastroenterology, Institute of Medical Sciences, Banaras Hindu University, Varanasi, Ultra Pradesh 221005, India

## Abstract

*Background*. Multifactorial scorings, radiological scores, and biochemical markers may help in early prediction of severity, pancreatic necrosis, and mortality in patients with acute pancreatitis (AP). *Methods*. BISAP, APACHE-II, MOSS, and SIRS scores were calculated using data within 24 hrs of admission, whereas Ranson and Glasgow scores after 48 hrs of admission; CTSI was calculated on day 4 whereas IL-6 and CRP values at end of study. Predictive accuracy of scoring systems, sensitivity, specificity, and positive and negative predictive values of various markers in prediction of severe acute pancreatitis, organ failure, pancreatic necrosis, admission to intensive care units and mortality were calculated. *Results*. Of 72 patients, 31 patients had organ failure and local complication classified as severe acute pancreatitis, 17 had pancreatic necrosis, and 9 died (12.5%). Area under curves for Ranson, Glasgow, MOSS, SIRS, APACHE-II, BISAP, CTSI, IL-6, and CRP in predicting SAP were 0.85, 0.75, 0.73, 0.73, 0.88, 0.80, 0.90, and 0.91, respectively, for pancreatic necrosis 0.70, 0.64, 0.61, 0.61, 0.68, 0.61, 0.75, 0.86, and 0.90, respectively, and for mortality 0.84, 0.83, 0.77, 0.76, 0.86, 0.83, 0.57, 0.80, and 0.75, respectively. *Conclusion*. CRP and IL-6 have shown a promising result in early detection of severity and pancreatic necrosis whereas APACHE-II and Ranson score in predicting AP related mortality in this study.

## 1. Introduction

Acute pancreatitis (AP) is defined as an inflammatory process of the pancreas with possible peripancreatic tissue and multiorgan involvement inducing multiorgan dysfunction syndrome (MODS) with an increased mortality rate [1]. The incidence of acute pancreatitis per 100,000 population ranges from 5 to 80 cases per year, with the highest incidence rates being seen in Finland and the USA [2].

According to the Atlanta Classification, severe acute pancreatitis (SAP) is defined as an AP associated with local and/or systemic complications. Atlanta classification is a clinically based classification defining AP, severity, and complications. Development of organ dysfunction within 72 h of symptom onset is defined as an early severe acute pancreatitis (ESAP). Early severe acute pancreatitis is characterized by a short course, progressive MODS, early hypoxemia, increased incidence of necrosis, infection, and abdominal compartment syndrome (ACS) [3]. Multiorgan dysfunction syndrome, the extent of pancreatic necrosis, infection, and sepsis are the major determinants of mortality in AP [4]. Pancreatic necrosis is considered as a potential risk for infection, which represents the primary cause of late mortality. Occurrence of acute respiratory (ARF), cardiovascular (CVF), and renal failures (RF) can predict the fatal outcome in SAP [5]. A wide range of mortality (20%–60%) has been reported in SAP. AP occurs when pancreatic enzymes are prematurely activated inside the pancreas leading to autodigestion of the gland and local inflammation [6]. These enzymes can also reach the bloodstream, stimulating the production of inflammatory cytokines and tumor necrosis factor-*α* (TNF-*α*) from leukocytes. The release of those substances triggers an inflammatory cascade, which leads to the SIRS [7]. Accurate diagnosis of SAP on admission to the hospital is of paramount importance and there is, therefore, agreement about the need for finding predictors of severe disease to identify patients who are at risk of morbidity and death.

Severe acute pancreatitis implies the presence of organ failure, local complications, or pancreatic necrosis and associated disruption of the pancreatic blood supply [8]. Several prognostic markers have been developed for severity stratification in acute pancreatitis. Multifactorial scoring systems incorporating clinical and biochemical criteria for severity assessment have been in use for some decades. These include the 11 criteria described by Ranson et al. in the 1970s [9], the Glasgow score (eight criteria), [10], MOSS score (12 criteria), BISAP score (5 criteria), and the acute physiology and chronic health evaluation (APACHE II) score (14 criteria) [11]. The sensitivity and specificity of these scoring systems for predicting severe acute pancreatitis range between 55% and 90%, depending on the cut-off number and the timing of scoring [12]. Limitations of these scoring systems have been either the inability to obtain a complete score until at least 48 hours into the illness (Ranson and Glasgow scores) or the complexity of the scoring system itself (APACHE II). The APACHE-II score has not been developed specifically for acute pancreatitis but has been proven to be an early and reliable tool.

Regarding imaging dynamic contrast-enhanced CT (DCT), it is the imaging modality of choice for staging acute pancreatitis and for detecting complications [13]. DCT has been shown to detect pancreatic parenchymal necrosis with a diagnostic sensitivity of 87% and an overall detection rate of 90% [13]. The morphologic severity of acute pancreatitis can be determined using a CT severity index (CTSI) that was developed by Balthazar and coworkers and then simplified and extended to monitor organ failure by Silverman, Banks, and colleagues in 2004 [13]. Comparison of the original CTSI with mortality showed a good correlation between higher CTSI values and mortality and morbidity, and this holds true for the modified CTSI. Furthermore, the modified CTSI correlates well with the length of hospital stay and the development of organ failure [13].

Among single biochemical markers, C-reactive protein (CRP) remains the most useful. Despite its delayed increase, peaking not earlier than 72 h after the onset of symptoms, it is accurate and widely available. As a single prognostic marker, an elevated C-reactive protein (CRP) concentration of greater than 150 mg/L indicates that acute pancreatitis has a complicated course with a sensitivity of 85% in the first 72 h after the onset of symptoms. Although detection of elevated CRP levels is sensitive for severe acute pancreatitis it is not specific for the disease, and other causes of inflammation such as cholangitis and pneumonia need to be ruled out before severity assessment by measurement of CRP [14]. Among them the proinflammatory cytokine interleukin 6 (IL-6) seems to be the most promising parameter for use in clinical routine. It has been proven to be significantly increased in severe acute pancreatitis in comparison with mild disease already on the day of admission to hospital [15], with a peak concentration on day 3 after the clinical onset of the disease, and therefore to be helpful for early severity stratification. Sensitivities of 69–100% with specificities in the range 70–86% for the detection of severe acute pancreatitis are reported [15]. As the concentrations increase earlier than those of acute-phase proteins, several clinical studies have addressed the usefulness of the early prediction of severe acute pancreatitis, resulting in promising results for interleukin 6. Procalcitonin (PCT) is another marker that has been evaluated as a prognostic indicator for pancreatitis. Procalcitonin, the biologically inactive propeptide of calcitonin, is a more rapid acute-phase reactant with the ability to indicate a status of bacterial or fungal infection and sepsis. Several studies have indicated its diagnostic value for the differentiation between mild and severe acute pancreatitis within the first 24 h of disease presentation [16], showing a sensitivity of 89% and a specificity of 82%, in a recent meta-analysis, but with a significant heterogeneity between individual studies [17].

## 2. Material and Methods

The aim of this prospective study was to study the various prognostic markers like Ranson, Glasgow, APACHE II, MOSS, SIRS, BISAP, CTSI, IL-6, CRP, and procalcitonin in cases of acute pancreatitis and to analyze the comparison between various prognostic markers in prediction of severe acute pancreatitis (SAP), organ failure (OF), pancreatic necrosis (PNec), length of hospital stay (LOHS), requirement of ICU admission (ICUA), and mortality in acute pancreatitis.

After approval by the Institutional Review Board, this prospective study included 72 patients who were clinically suspected to have acute pancreatitis in a single surgical unit in Department of General Surgery, IMS, BHU, in collaboration with the Department of Gastroenterology, Department of Pathology, Department of Radiology, and Causality services from July 2010 to July 2012. Informed and written consent was obtained from all patients. The diagnosis of acute pancreatitis (AP) was based on the presence of two of the following three features: (1) abdominal pain characteristic of AP, (2) serum amylase and/or lipase ≥3 times the upper limit of normal, and (3) characteristic finding of AP on abdominal CT Scan.

Demographic, radiographic, and laboratory data were collected from all these patients. In all these patients the following prognostic markers were used to know the severity of the disease (SAP), pancreatic necrosis (PNec), requirement of ICU admission, length of hospital stay (LOHS), and mortality: (1) Ranson score, (2) Glasgow score, (3) MOSS score, (4) SIRS score, (5) APACHE II score, (6) BISAP score, (7) CTSI score, (8) IL-6, (9) CRP, and (10) procalcitonin. BISAP score, APACHE II score, and multiple organ system score (MOSS) were calculated using data from the first 24 hours of admission and Ranson and Glasgow scores were calculated using data in first 24 hours and after 48 hours of admission. Presence of features of systemic inflammatory response syndrome (SIRS) was noted within the first 24 hours of admission. 2 mL of blood sample was collected on day 1 for IL-6. Serum was extracted after centrifugation in the Department of Pathology and stored at −72°C. Another 2 mL of blood sample was collected for procalcitonin card test. It is a semiquantitative method for rapid calculation of procalcitonin value using B.R.A.H.M.S. PCT-Q card. For this, serum was extracted from the blood sample after centrifugation. One drop of serum was put into the card and reading was taken after 30 minutes. Color of the test bar was matched with color given on the card. Value was noted according to the colour coding. Value ranging from <0.5 ng/mL up to >10 ng/mL. >0.5 ng/mL is taken as the cut-off value for detection of severity of acute pancreatitis according to the previous literature. This test was done in 42 cases only. On day 2 another 2 mL of blood sample was collected for C-reactive protein (CRP) and stored as serum at −72°C along with the samples of IL-6 to determine the value at the end of the study. CECT was performed in required cases on day 4 to look for pancreatic necrosis (PNec), local complications, and possible aetiology of AP. CTSI score was noted after CT scan.

Patients were classified as mild AP and severe AP, based on the presence of organ failure for more than 48 hrs and local complications. Organ failure included shock (systolic blood pressure < 90 mmHg), pulmonary insufficiency (arterial PO_2_ < 60 mmHg at room air or the need for mechanical ventilation), or renal failure (serum creatinine level > 2 mg/dL after rehydration or hemodialysis). PNec was assessed by CECT; evidence of PNec on CT was defined as lack of enhancement of pancreatic parenchyma with contrast. At the end of the study, values of IL-6 and CRP were calculated using ELISA kit.

### 2.1. Statistics

Normally distributed continuous variables were expressed as means. At the selected cut-off scores, each predictive system was evaluated for significant relationship to the severity, organ failure, pancreatic necrosis, need for ICU admission, and mortality by two-by-two contingency tables. The diagnostic cut-off value was expressed as its sensitivity, specificity, positive predictive value, negative predictive value, accuracy, and the area under the curve (AUC) under the receiver-operator characteristic (ROC) curve. The predictive accuracy of each scoring system and biochemical marker was measured by the area under the receiver-operating curve (AUC). All statistical analysis was made with SPSS software version 16.

## 3. Observation and Results

### 3.1. Patient's Characteristics

Mean age of presentation was 40.5 years (range 18–76) with 51.4% males and 91.7% Hindu. The etiologies of AP included biliary (64%), alcoholic (13%), idiopathic (9%), hypertriglyceridemia (2%), post-ERCP (2%), and trauma (2%) (Table 1).

Thirty-one patients (43.1%) were diagnosed as having SAP (organ failure with local complications), twenty five patients (34.7%) developed persistent organ failure, and seventeen patients (23.6%) had evidence of pancreatic necrosis on CECT. The average length of hospital stay was 10 days. Nine patients (12.5%) needed ICU admission and nine patients (12.5%) died during hospitalization. 54 patients (75%) underwent CECT abdomen on day-4. IL-6 and CRP were done in 60 patients of whom 46.7% had IL-6 value of ≥50 pg/mL and 41.7% of cases had CRP value of ≥150 mg/L.  Table 2 shows patient characteristics of the study cohort. 

### 3.2. Comparison of Scoring Systems in Predicting SAP, Organ Failure, Pancreatic Necrosis, Length of Hospital Stay, ICU Admission, and Mortality

In prediction of SAP according to the AUC (with 95% CI) CRP (0.91 (0.83–0.99)) and IL-6 (0.90 (0.81–0.99)) had the highest accuracy, followed by APACHE II (0.88 (0.79–0.97)) and Ranson (0.85 (0.76–0.92)). Also for prediction of pancreatic necrosis according to AUC (with 95% CI) CRP (0.90 (0.82–0.97)) and IL-6 (0.86 (0.77–0.94)) had the highest accuracy as compared to other markers, followed by CTSI (0.75 (0.59–0.91)) and for prediction of mortality according to AUC (with 95% CI) accuracy was highest for APACHE II 0.86 (0.77–0.95) followed by Ranson score (0.84 (0.75–0.94)). AUCs for each scoring system in predicting SAP, PNEC, and mortality are shown in Table 3. Among the various markers CRP and IL-6 had the highest accuracy in predicting both SAP and PNEC but for mortality APACHE II and Ranson showed a little higher accuracy than the above two markers. 

**Figure 1:**
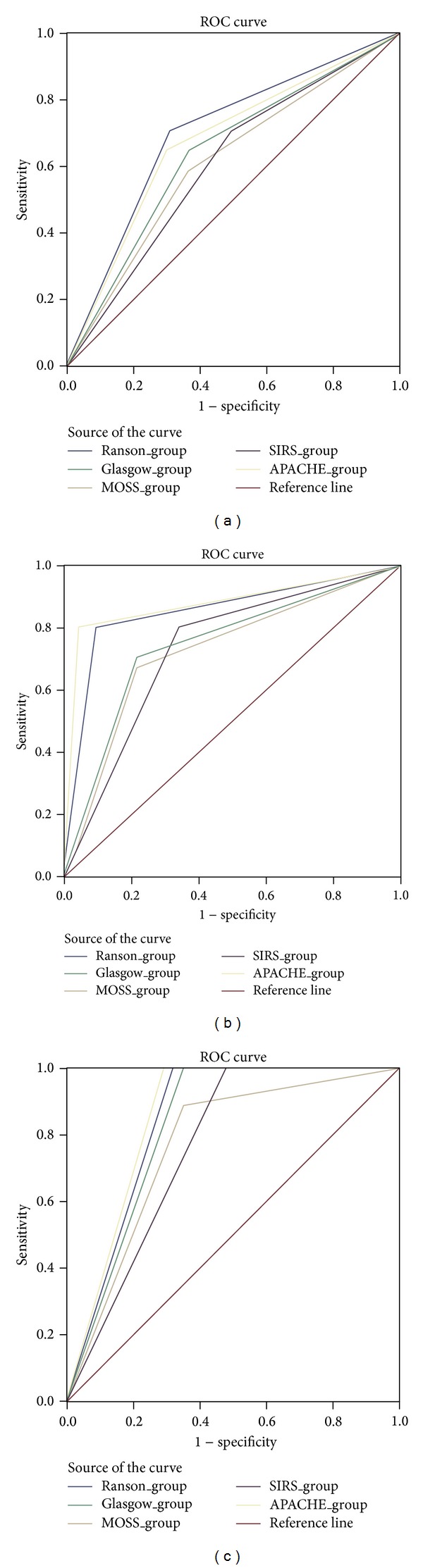
AUC comparison of various scoring systems in predicting SAP (a), pancreatic necrosis (b), and mortality (c). Diagonal segments are produced by ties.

Among the multifactorial scoring systems, APACHE II and Ransons score had highest accuracy for predicting SAP and PNEC (Figures 1(a) and 1(c)). CTSI score as expected had the highest accuracy for prediction of pancreatic necrosis among the scoring systems (Figure 1(b)).

On the basis of the highest sensitivity and specificity values generated from the receiver-operating characteristic curves, the following cut-offs were selected for further analysis. Ranson ≥ 3, Glasgow ≥ 3, MOSS ≥ 5, BISAP ≥ 2, APACHE II ≥ 8, CTSI ≥ 5, procalcitonin ≥ 0.5 ng/mL, CRP ≥ 150 mg/L, and IL-6 ≥ 50 pg/mL. The observed incidence of severe disease, organ failure, pancreatic necrosis, need for ICU admission, average length of hospital stay, and mortality stratified by the various markers with their cut-offs is given in the Table 4. The number of patients with Ranson score ≥ 3 was 35, Glasgow ≥ 3 was 31, MOSS ≥ 5 was 42, APACHE II ≥ 8 was 32, SIRS was 39, BISAP ≥ 2 was 36, procalcitonin ≥ 0.5 ng/mL was 24, CRP ≥ 150 mg/L was 25, and IL-6 ≥ 50 pg/mL was 28.

IL-6, CRP, and procalcitonin have the highest sensitivity for prediction of SAP. The specificity, PPV, NPV, and accuracy of IL-6 and CRP are also very high for prediction of SAP. Regarding OF the sensitivity for prediction is very high for procalcitonin, IL-6, APACHE II, and Ransons score and CRP is more specific and more accurate in prediction of OF. CRP is highly sensitive and specific for prediction of PNec with a very high accuracy. IL-6 and CTSI scores are the next markers which had a very high sensitivity for prediction of PNec. There was a higher need of ICU admission in patients with SIRS and a high MOSS, APACHE and Ranson scores. Regarding mortality, multifactorial scoring systems (APACHE II, Ranson, SIRS, and Glasgow), procalcitonin, and IL-6 were more accurate in predicting mortality. IL-6 and APACHE II were more accurate in predicting mortality (Table 5).

## 4. Discussion

In this study we have compared all the scoring systems, biochemical and radiological markers for prediction of morbidity and mortality in acute pancreatitis. We confirmed that single biochemical markers can be used as a reliable indicator for early stratification of severity of acute pancreatitis within 24 hours of admission. 

The overall mortality in our cohort was 12.5% and 43.1% of patients had SAP. As expected the proportion of patients with severe disease and mortality in our cohort was higher as compared to previous studies [18]; this is probably because of a more number of referred cases admitted in our hospital. 

Ranson's score is composed of 11 measures that are recorded as binary values on admission and at 48 hrs, and its primary aim was to evaluate the function of early operative intervention in patients with AP. A composite score of 3 or more is commonly used to classify a patient as having severe disease. Studies confirmed sensitivity from 40% to 90% [19]. Glasgow score proposed by Imrie for both alcohol and biliary acute pancreatitis seems to be more precise than that of Ranson, with a sensitivity for the assessment of severe acute pancreatitis of 56%–85% [20] using 8 laboratory factors within the first 48 h of treatment to calculate it [10], and more than three positive criteria indicate severe acute pancreatitis. Though fewer markers are taken into account, this score as well as the Ranson score predicts severe acute pancreatitis. Another commonly used severity index is the APACHE II index [21, 22]. This clinical tool measures the physiological response to injury and inflammation-driven stress and was initially designed to predict prolonged intensive care unit treatment and mortality. Papachristou et al. [18] found sensitivity, specificity, and accuracy of 84.2%, 89.8%, and 94% of Ranson criteria for prediction of SAP and 70.3%, 71.9% and 78% for APACHE II score. In our study we have found sensitivity, specificity, and accuracy of 83.9%, 78%, and 85% of Ranson criteria for prediction of SAP and 80.6%, 82.9%, and 88% for APACHE II score. Similar result has been found for prediction of pancreatic necrosis and mortality. For Glasgow score we found 71%, 78%, and 75% sensitivity, specificity and accuracy for prediction of SAP, respectively, which is in accordance with the study done by Blamey et al. [10]. 

BISAP and MOSS scores are newly developed prognostic scoring systems containing data that are frequently evaluated at the time of admission which are accurate in predicting patient's outcome [23]. BISAP and MOSS scores have the advantage over Ranson and Glasgow scores of being calculated within 24 hrs of admission. BISAP score is higher in patients having SIRS, in older patients and in patients with altered mental status, whereas Ranson score seems to perform accurate prediction of persistent organ failure (sensitivity 92%, specificity 74.5%, PPV 65.7%, and NPV 94.6%). BISAP has the disadvantage that it cannot easily distinguish transient from persistent organ failure. SIRS is one of the leading events responsible for the mortality of AP. In our study we have a sensitivity of 80.6%, 84%, and 100% for prediction of SAP, OF, and mortality and an accuracy of 73%, 61%, and 76%, respectively.

Computed tomography severity index has shown a strong positive correlation with the development of complications and mortality in patients with AP [12, 24]. It was developed by Balthazar et al. to evaluate the degree of pancreatic edema, necrosis and the presence of peripancreatic fluid collections. In the CTSI pilot study, a score of 7–10 was able to predict 92% morbidity and 17% mortality rate in patients with AP, compared to the low morbidity (2%) and mortality (0%) associated with a CTSI score of 0-1 [25]. In our study we have found that CTSI had the highest sensitivity of 87.5% and 91.3% NPV among multifactorial scoring system in prediction of pancreatic necrosis as expected and the lowest sensitivity in prediction of organ failure (65.2%).

C-Reactive protein (CRP) is an acute phase reactant produced by the liver in response to interleukin-1, interleukin-6, and tumor necrosis factor-*α* and it is the most widely available, low-cost, and well-studied marker of severity in AP. A cut-off level of 150 mg/L within the first 48 hrs of symptom onset has sensitivity and specificity of 80–86% and 61–84%, respectively, for SAP and accuracy > 80% for necrotizing pancreatitis [26], CRP was done in 60 patients in our study; 58.3% of patients were having CRP value < 150 mg/L whereas 41.7% of cases had value of ≥150 mg/L. In patients having CRP level > 150 mg/L incidence of SAP, OF, PNEC, ICUA, MORT, and LOHS was found to be 100% (25), 76.0% (19), 68.0% (17), 24.0% (6), and 24.0% (6) with an average length of hospital stay of 13.8 days, respectively. In our study CRP had the highest sensitivity (100%), NPV (100%), and specificity (81.4%) for pancreatic necrosis, followed by sensitivity of 86.2% and specificity and PPV of 100% for prediction of SAP. As a whole CRP is a good marker for prediction of complications and mortality in acute pancreatitis. The AUC for prediction for PNec was higher for CRP 0.90 (0.82–0.77). 

Activated leukocytes release proinflammatory cytokines that stimulate the liver to produce acute phase proteins. Since the concentration of cytokines increases before acute phase proteins, numerous clinical studies have been done to assess the usefulness of cytokines, such as interleukin-(IL-) 1, IL-6, IL-8, IL-10, and IL-18, in predicting severity early in the course of AP. Most trials have focused on the proinflammatory cytokines IL-6. Value of IL-6 is significantly elevated in SAP on the day of admission and tends to peak at 72 hrs after the clinical onset of disease, which makes IL-6 an excellent marker of early severity stratification. A 2009 meta-analysis, defining severity by the Atlanta Classification, revealed that the sensitivity and specificity ranges for IL-6 in the first three days of admission were 81–83.6% and 75.6–85.3%, respectively, with an IL-6 AUC of 0.75 on day one and 0.88 on the second day of admission [24]. In our study IL-6 was done in 60 patients. 53.3% of patients were having IL-6 value <50 pg/mL and 46.7% were having value of ≥50 pg/mL. In patients having IL-6 level >50 pg/mL incidence of SAP, OF, PNEC, ICUA, MORT, and average LOHS found was to be 96.4% (27), 78.6% (22), 57.1% (16), 25.0% (7), and 32.1% (9) with an average length of hospital stay of 13.7 days, respectively. IL-6 has the highest sensitivity for prediction of SAP (93.1%), organ failure (95.7%), pancreatic necrosis (94.1%), and mortality (100%). Regarding specificity it has the highest specificity (96.8%) for SAP. It has very high NPV (93.8%) and accuracy (95.0%) for prediction of SAP. It has very high NPV (100%) for mortality and NPV (96.9%) for prediction of pancreatic necrosis.

Procalcitonin (PCT) is a propeptide of the hormone calcitonin, which is released by hepatocytes, peripheral monocytes, and G-cells of the thyroid gland. PCT level can be measured by a semiquantitative strip test for fast results or by a fully automated assay to obtain a more accurate measurement. An increased PCT level has been found to be an early predictor of severity [27–29], pancreatic necrosis, and organ failure [30] in patients with AP. In a recent meta-analysis a subgroup of 8 studies using PCT cut-off values of 0.5 ng/mL as a discriminator found that the sensitivity and specificity of PCT for development of SAP were 73% and 87%, respectively, with an overall AUC of 0.88 [31]. In our study procalcitonin has 100% sensitivity for prediction of organ failure and mortality with a sensitivity of 86.4% for prediction of SAP.

At the end of the study we found that for prediction of SAP IL-6 had the highest sensitivity of 93.1%, followed by CRP (86.2%), procalcitonin (86.4%), and Ranson score (83.9%). CRP had the highest specificity of 100% with an accuracy of 95%, followed by IL-6 (96.4%) with an accuracy of 95%. Ranson and APACHE II scores come as the next best predictors of SAP. For prediction of organ failure procalcitonin had the highest sensitivity and NPV of 100%, followed by IL-6, Ranson, and APACHE II scores. Accuracy for prediction of organ failure is the highest for procalcitonin (83.3%) and APACHE II scores (83.3%). For prediction of pancreatic necrosis it is the CRP which has the maximum sensitivity and NPV of 100%, followed by IL-6 which has a sensitivity of 94.1% and NPV of 96.9%. Ranson and MOSS scores are the next best predictors of pancreatic necrosis. CTSI has a sensitivity of 87.5% for prediction of pancreatic necrosis. The AUC for pancreatic necrosis is the highest for CRP (0.90), followed by IL-6 (0.86), and Ranson score (0.70). Requirement of ICU admission was best predicted by MOSS score and SIRS with 100% sensitivity and NPV, followed by Ranson and Glasgow. Glasgow score has the highest accuracy for need of ICU requirement (66.7%). For the mortality predictors it is IL-6, procalcitonin, Ranson, Glasgow, APACHE II, MOSS, and SIRS which have 100% sensitivity and NPV; AUC for mortality prediction is the highest for Ranson (0.84) and APACHE II scores (0.86).

## 5. Conclusion

We conclude that determining the serum concentration of IL-6 on the first day and/or together with serum CRP concentration on the 2nd day of admission is helpful in earlier prediction and assessment of the severity of acute pancreatitis taking into consideration the disadvantages of multifactorial scoring systems. However, there is no ideal single method in assessing the severity of the disease. Individual preference and available institutional facilities influence the method chosen for prognostic assessment of acute pancreatitis.

## Figures and Tables

**Table 1:** Etiology of acute pancreatitis.

Etiology	No. of cases	Percentage
Biliary	44	61.1
Alcoholic	13	18.0
Idiopathic	9	12.5
Hypertriglyceridemia	2	2.8
Post-ERCP	2	2.8
Traumatic	2	2.8

ERCP: endoscopic retrograde cholangiopancreatography.

**Table 2:** Patients characteristics.

Patients characteristics	No. of cases	Percentage
Sex		
Male	37	51.4
Female	35	48.6
Religion		
Hindu	66	91.7
Muslim	6	8.3
Age group		
11–20	9	12.5
21–30	17	23.6
31–40	12	16.7
41–50	16	22.2
51–60	5	6.9
61–70	13	18.1
Comorbidities		
Diabetes mellitus	3	4.2
Hypertension	1	1.4
Other	2	2.8
No comorbid condition	62	86.1
Both DM and hypertension	4	5.6
BMI		
<18.5	1	1.4
18.5–24.9	55	76.4
25–29.9	14	19.4
30–34.9	2	2.8
Presentations		
Pain abdomen	72	100
Radiating	62	86.1
Nonradiating	10	13.9
Peritonitis	62	86.1
Localized	36	58.1
Diffuse	26	41.9
Nausea	6	8.3
Vomiting	51	70.8
Distension abdomen	29	40.2
Nonpassage of flatus and stool	21	29.2
Breathlessness	22	30.6

**Table 3:** AUC (area under curve) of different prognostic markers in predicting SAP, PNEC, and mortality.

AUC (95% CI)	SAP	PNEC	Mortality
Ranson	0.85 (0.76–0.92)	0.70 (0.55–0.89)	0.84 (0.75–0.94)
Glasgow	0.75 (0.63–0.86)	0.64 (0.49–0.79)	0.83 (0.73–0.93)
MOSS	0.73 (0.61–0.85)	0.61 (0.46–0.77)	0.77 (0.62–0.92)
SIRS	0.73 (0.61–0.85)	0.61 (0.46–0.76)	0.76 (0.64–0.88)
APACHE II	0.88 (0.79–0.97)	0.68 (0.58–0.83)	0.86 (0.77–0.95)
BISAP	0.80 (0.71–0.91)	0.61 (0.47–0.72)	0.83 (0.69–0.97)
IL-6	0.90 (0.81–0.99)	0.86 (0.77–0.94)	0.80 (0.69–0.91)
CRP	0.91 (0.83–0.99)	0.90 (0.82–0.97)	0.75 (0.63–0.88)
CTSI	0.66 (0.53–0.79)	0.75 (0.59–0.91)	0.57 (0.35–0.78)

MOSS: multiple organ system score, APACHE II: acute physiology and chronic health evaluation II, SIRS: systemic inflammatory response syndrome, BISAP: bedside index for severe acute pancreatitis, IL-6: interleukin 6, CRP: C-reactive protein, and CTSI: CT severity index.

**Table 4:** Incidence of SAP, OF, PNec, ICUA, and mortality stratified by Ranson, Glasgow, MOSS, APACHE II, SIRS, BISAP scores, Procalcitonin, IL-6, and CRP.

Markers	No. (%)	%SAP (*n*)	%OF (*n*)	%PNec (*n*)	%ICUA (*n*)	%MORT (*n*)	LOHS (days)
Ranson							
<3	37 (51.4)	13.5 (5)	5.4 (2)	13.5 (5)	2.7 (1)	0 (0)	8.24
≥3	35 (48.6)	74.3 (26)	65.7 (23)	34.3 (12)	22.9 (8)	25.7 (9)	12.08
Total	72 (100)	43.1 (31)	34.7 (25)	23.6 (17)	12.5 (9)	12.5 (9)	10.1
Glasgow							
<3	41 (56.9)	22.0 (9)	14.6 (6)	14.6 (6)	2.4 (1)	0 (0)	8.14
≥3	31 (43.1)	71.0 (22)	61.3 (19)	35.5 (11)	25.8 (8)	29.0 (9)	12.7
Total	72 (100)	43.1 (31)	34.7 (25)	23.6 (17)	12.5 (9)	12.5 (9)	10.1
MOSS							
<5	30 (41.7)	23.3 (7)	13.3 (4)	16.7 (5)	0 (0)	0 (0)	8.5
≥5	42 (58.3)	57.1 (24)	50.0 (21)	28.6 (12)	21.4 (9)	21.4 (9)	11.23
Total	72 (100)	43.1 (31)	34.7 (25)	23.6 (17)	12.5 (9)	12.5 (9)	10.1
APACHE II							
<8	40 (55.6)	15.0 (6)	2.5 (1)	15.0 (6)	2.5 (1)	0 (0)	8.22
≥8	32 (44.4)	78.1 (25)	75.0 (24)	34.4 (11)	25.0 (8)	28.1 (9)	12.48
Total	72 (100)	43.1 (31)	34.7 (25)	23.6 (17)	12.5 (9)	12.5 (9)	10.1
SIRS							
Absent	33 (45.8)	18.2 (6)	12.1 (4)	15.2 (5)	0 (0)	0 (0)	7.84
Present	39 (54.2)	64.1 (25)	53.8 (21)	30.8 (12)	23.1 (9)	23.1 (9)	12.02
Total	72 (100)	43.1 (31)	34.7 (25)	23.6 (17)	12.5 (9)	12.5 (9)	10.1
BISAP							
<2	36 (50)	22.2 (8)	13.9 (5)	19.4 (7)	5.6 (2)	2.7 (1)	7.5
≥2	36 (50)	63.9 (23)	55.6 (20)	27.8 (10)	19.4 (7)	22.8 (8)	10.2
Total	72 (100)	43.1 (31)	34.7 (25)	23.6 (17)	12.5 (9)	12.5 (9)	10.1
CTSI							
<5	23 (42.6)	39.1 (9)	34.8 (8)	17.4 (4)	13.0 (3)	8.7 (2)	8.6
≥5	31 (57.4)	54.8 (17)	41.9 (13)	38.7 (12)	12.9 (4)	16.1 (5)	11.22
Total	54 (100)	48.1 (26)	38.9 (21)	29.6 (16)	13.5 (7)	13.5 (7)	9.91
Procalcitonin							
<0.5 ng/mL	18 (42.9)	16.7 (3)	0 (0)	16.7 (3)	5.6 (1)	0 (0)	9.19
>0.5 ng/mL	24 (57.1)	79.7 (19)	70.8 (17)	45.8 (11)	20.8 (5)	29.2 (7)	11.5
Total	42 (100)	52.4 (22)	40.5 (17)	33.3 (14)	14.3 (6)	16.7 (7)	10.92
IL-6							
<50 pg/mL	32 (53.3)	6.2 (2)	3.1 (1)	3.1 (1)	3.1 (1)	0 (0)	7.68
≥50 pg/mL	28 (46.7)	96.4 (27)	78.6 (22)	57.1 (16)	25.0 (7)	32.1 (9)	13.7
Total	60 (100)	48.3 (29)	38.3 (23)	23.6 (17)	13.3 (8)	12.5 (9)	10.69
CRP							
<150 mg/L	35 (58.3)	11.4 (4)	11.4 (4)	0 (0)	5.7 (2)	8.6 (3)	8.54
≥150 mg/L	25 (41.7)	100 (25)	76.0 (19)	68.0 (17)	24.0 (6)	24.0 (6)	13.28
Total	60 (100)	48.3 (29)	38.3 (23)	23.6 (17)	13.3 (8)	12.5 (9)	10.91

SAP: severe acute pancreatitis, OF: organ failure, PNec: pancreatic necrosis, ICUA: intensive care unit admission, LOHS: length of hospital stay, MOSS: multiple organ system score, APACHE II: acute physiology and chronic health evaluation II, SIRS: systemic inflammatory response syndrome, BISAP: bedside index for severe acute pancreatitis, IL-6: interleukin 6, CRP: C-reactive protein, and CTSI: CT severity index.

**Table 5:** Sensitivity, specificity, PPV, NPV, and accuracy of different markers in predicting SAP, OF, PNec, need for ICU admission, and mortality.

	Sensitivity	Specificity	PPV	NPV	Accuracy	Kappa (95% CI)
Severe acute pancreatitis						
Ranson	83.9	78.0	74.3	86.5	80.6	0.61 (0.40–0.75)
Glasgow	71.0	78.0	71.0	78.0	75.0	0.49 (0.27–0.66)
MOSS	77.4	56.1	57.1	76.7	65.3	0.32 (0.10–0.49)
APACHE II	80.6	82.9	78.2	85.0	81.9	0.63 (0.42–0.78)
SIRS	80.6	65.9	64.1	81.8	72.2	0.45 (0.23–0.61)
BISAP	74.2	68.3	63.4	77.8	70.8	0.42 (0.19–0.59)
CTSI	65.4	50.0	54.8	60.9	57.4	0.15 (−0.11–0.39)
IL-6	93.1	96.8	96.4	93.8	95.0	0.90 (0.73–1.0)
CRP	86.2	100	100	88.6	93.3	0.87 (0.70–0.87)
Procalcitonin	86.4	75.0	79.2	83.3	81	0.62 (0.33–0.79)

Organ failure						
Ranson	92.0	74.5	65.7	94.6	80.6	0.61 (0.42–0.69)
Glasgow	76.0	74.5	61.3	85.4	75.0	0.48 (0.26–0.64)
MOSS	84.0	55.3	50.0	86.7	65.3	0.34 (0.14–0.46)
APACHE II	96.0	60.9	49.0	97.5	70.8	0.44 (0.30–0.48)
SIRS	84.0	61.7	53.8	87.9	69.4	0.40 (0.20–0.53)
BISAP	80.0	66.0	55.6	86.1	70.8	0.42 (0.20–0.56)
CTSI	65.2	45.5	45.5	65.2	53.6	0.10 (−0.15–0.32)
IL-6	95.7	33.3	78.6	75.0	78.1	0.35 (0.03–0.50)
CRP	82.6	83.8	76.0	88.6	83.3	0.65 (0.42–0.80)
Procalcitonin	100	72.0	70.8	100	83.3	0.68 (0.45–0.68)

Pancreatic necrosis						
Ranson	70.6	58.2	34.3	86.5	61.1	0.21 (0.01–0.36)
Glasgow	64.7	63.6	35.5	85.4	63.9	0.22 (0.01–0.39)
MOSS	70.6	45.5	28.6	83.3	51.4	0.11 (−0.07–0.24)
APACHE II	64.7	61.8	34.4	85.0	62.5	0.20 (−0.004–0.37)
SIRS	70.6	50.9	30.8	84.8	55.6	0.15 (−0.04–0.29)
BISAP	58.8	52.7	27.8	80.6	54.2	0.08 (−0.11–0.25)
CTSI	87.5	55.3	45.2	91.3	57.4	0.20 (−0.31–0.36)
IL-6	94.1	72.1	57.1	96.9	78.3	0.55 (0.35–0.61)
CRP	100	81.4	68.0	100	86.7	0.71 (0.53–0.71)
Procalcitonin	78.6	53.6	45.8	83.3	61.9	0.27 (0.003–0.85)

ICU admission						
Ranson	88.9	57.1	22.9	97.3	61.1	0.21 (0.45–0.25)
Glasgow	88.9	63.5	25.8	97.6	66.7	0.26 (0.09–0.31)
MOSS	100	47.6	21.4	100	54.2	0.19 (0.06–0.19)
APACHE II	88.9	61.9	25.0	97.5	65.3	0.24 (0.08–0.29)
SIRS	100	52.4	23.1	100	58.3	0.22 (0.09–0.21)
BISAP	77.8	54.0	19.4	94.4	56.9	0.14 (−0.01–0.20)
CTSI	87.5	55.3	45.2	91.3	64.8	0.001 (−0.15–0.12)
IL-6	87.5	59.6	25.0	96.9	63.3	0.23 (0.05–0.29)
CRP	75.0	63.5	24.0	94.3	65.0	0.20 (0.01–0.31)
Procalcitonin	83.3	47.2	20.8	94.4	52.4	0.14 (−0.06–0.21)

Mortality						
Ranson	100	58.7	25.7	100	63.9	0.26 (0.12–0.26)
Glasgow	100	65.7	29.0	100	64.4	0.32 (0.16–0.32)
MOSS	100	47.6	21.4	100	54.2	0.19 (0.06–0.19)
APACHE II	100	63.5	28.1	100	68.1	0.30 (0.15–0.30)
SIRS	100	52.4	23.1	100	58.3	0.22 (0.09–0.21)
BISAP	88.9	55.6	22.2	97.2	59.7	0.19 (0.04–0.24)
CTSI	71.4	44.7	16.1	91.3	48.1	0.07 (−0.10–0.16)
IL-6	100	62.7	32.1	100	68.3	0.34 (0.16–0.34)
CRP	66.7	62.7	24.0	91.4	63.3	0.17 (−0.03–0.31)
Procalcitonin	100	51.4	29.2	100	59.5	0.26 (0.07–0.26)

SAP: severe acute pancreatitis, OF: organ failure, PNec: pancreatic necrosis, ICUA: intensive care unit admission, LOHS: length of hospital stay, MOSS: multiple organ system score, APACHE II: acute physiology and chronic health evaluation II, SIRS: systemic inflammatory response syndrome, BISAP: bedside index for severe acute pancreatitis, IL-6; interleukin 6, CRP: C-reactive protein, and CTSI: CT severity index.
